# Low-yield respiratory sequencing in pediatric upper respiratory specimens: a case series and reporting framework

**DOI:** 10.3389/fped.2026.1865124

**Published:** 2026-08-05

**Authors:** Yunxia Xiu, Hua Shang, Chunna Ren, Xiaoyu Wang, Qingjun Li, Shiwei Zhang, He Wang, Hui Yue, Feipeng Zhao

**Affiliations:** 1 Second Clinical Medical School, Mudanjiang Medical University, Mudanjiang, China; 2 Department of Laboratory Medicine, The Second Affiliated Hospital of Mudanjiang Medical University, Mudanjiang, China; 3 First Clinical Medical School, Mudanjiang Medical University, Mudanjiang, China; 4 Department of Anesthesiology, Daqing People’s Hospital, Daqing, China; 5 Department of Neurosurgery, Daqing People’s Hospital, Daqing, China; 6 Department of Scientific Research, Mudanjiang Medical University, Mudanjiang, China; 7 School of Basic Medical Sciences, Mudanjiang Medical University, Mudanjiang, China

**Keywords:** clinical metagenomics, diagnostic discordance, diagnostic stewardship, low-yield sequencing, pediatric respiratory infection, reporting framework, upper respiratory specimens

## Abstract

Clinical interpretation of respiratory sequencing results is difficult when analytical support is sparse or when sequencing findings do not align with routine laboratory reports. We expanded an ultra-low-yield index case into a retrospective descriptive pediatric case series to characterize recurrent interpretive scenarios and support a pragmatic laboratory reporting framework. We retrospectively reviewed archived upper respiratory specimens from pediatric patients with respiratory symptoms who had undergone both routine respiratory testing and sequencing-based pathogen analysis. Clinical features, routine-test interpretation, sequencing metrics, top reported hits, result-return timing, management review, and short-term outcomes were abstracted from retrievable records. Ten children aged 6–10 years were included. Routine testing was classified as influenza-positive in 8 cases and negative in 2 cases. Retained pathogen-associated contigs were sparse (median: 12.5; range: 5–17), and mapped read support was low (median: 408.5 read pairs; range: 384–453). Top low-support hits included rhinovirus/rhinovirus B in 6 cases, respiratory syncytial virus in 2 cases, and Mycoplasma-related hits in 2 cases. The Mycoplasma-related findings were interpreted cautiously because limited report-level sequencing evidence and the absence of orthogonal confirmation, paired serology, lower-respiratory specimen confirmation, or specimen-matched negative-control review prevented confident distinction between active infection, carriage or colonization, transient detection, coinfection of uncertain relevance, and contamination. No case had documented orthogonal confirmation or a specimen-matched negative control. Provider-level clarification indicated the use of batch-level negative controls, the absence of respiratory pathogen-related background reads, contamination-aware filtering, and manual review, although raw batch-level quality-control (QC) reports were not independently retrievable. Low-yield respiratory sequencing results in this small, purposively selected pediatric series were best understood as analytically limited signals requiring cautious interpretation. Accordingly, these low-support detections should be treated as hypothesis-generating observations rather than disease-defining findings. The proposed framework should be interpreted as a preliminary reporting aid for structured interpretation, not as a validated diagnostic algorithm.

## 1 Introduction

Sequencing-based respiratory pathogen detection has increasing clinical appeal when routine assays are unavailable, incomplete, or difficult to reconcile with the clinical picture (1, 2). However, upper respiratory specimens often contain limited pathogen nucleic acid relative to host and background material, yielding fragmented assemblies, sparse contig-level evidence, and uncertain taxonomic interpretation (3, 4). Studies of pediatric respiratory metagenomic next-generation sequencing (mNGS) have shown potential value in children with community-acquired, severe, or refractory pneumonia, particularly when lower respiratory specimens such as bronchoalveolar lavage fluid are available and results can be interpreted alongside conventional microbiological testing (13, 14). However, those studies do not remove the interpretive challenges posed by low-yield upper respiratory specimens, where low microbial biomass, background nucleic acid, asymptomatic viral detection, prolonged shedding, colonization or carriage, and contamination may complicate causal attribution (11, 12, 15–21). In our initial index case, a pediatric throat swab generated 50,445,331 raw read pairs, but only 13 pathogen-associated contigs were retained and 417 read pairs mapped to retained pathogen-associated contigs overall; two retained contigs showed high nucleotide identity to Rhinovirus B, yet influenza reverse-transcription polymerase chain reaction (RT-PCR) testing was unavailable and no specimen-matched negative control accompanied the sample. That case therefore supported cautious, graded interpretation and reporting rather than definitive etiologic attribution.

To explore whether that scenario reflected a broader real-world interpretive problem, we assembled a small pediatric case series of upper respiratory specimens with sequencing-based testing and clinically challenging relationships to routine laboratory reports. This study was not designed to establish diagnostic accuracy, estimate the prevalence of low-yield sequencing findings, or validate a diagnostic algorithm. Instead, it aimed to describe recurrent analytical and clinical features of low-yield respiratory sequencing results and to propose a preliminary pragmatic reporting framework for communicating low-support sequencing findings in pediatric respiratory diagnostic practice (3, 5). In this setting, the central question is not definitive pathogen assignment, but how low-support sequencing findings should be interpreted and communicated when routine laboratory results are discordant, incomplete, or difficult to reconcile.

## 2 Methods

### 2.1 Study design and case selection

This study was designed as a retrospective, clinically triggered descriptive case series rather than a consecutive cohort, diagnostic accuracy study, or prevalence-estimating study. Cases were identified between November 2025 and December 2025 from pediatric patients with respiratory symptoms who had undergone initial routine clinical and laboratory evaluation followed by treatment based on the first available assessment, but whose clinical response was considered suboptimal. Upper respiratory swab specimens had initially been collected for routine respiratory testing. After routine testing, the corresponding swab specimens were frozen and archived. When clinical response was considered suboptimal, the corresponding archived swab specimens were retrieved from storage and submitted for supplementary sequencing-based pathogen assessment to further clarify potential infectious etiologies.

The decision to obtain sequencing was based on unsatisfactory clinical response after initial evaluation and treatment, rather than on prior knowledge that the specimens would yield low-support or discordant sequencing results. The included cases were not consecutive cases of pediatric respiratory infection and should not be interpreted as representing all pediatric respiratory infections, all children undergoing routine respiratory testing, or all respiratory sequencing tests during the same period. Rather, they represent all archived upper respiratory swab specimens retrieved for this supplementary sequencing series during the defined study period. Cases were not selected on the basis of mapped read counts, retained contig numbers, top-hit identity, aligned length, or any predefined sequencing-metric pattern.

Cases were eligible for inclusion if they met all of the following criteria: pediatric age; respiratory symptoms; initial routine clinical or laboratory assessment followed by treatment with suboptimal clinical response; an archived corresponding upper respiratory swab specimen originally collected for routine respiratory testing and subsequently retrieved for supplementary sequencing-based pathogen analysis; archived routine respiratory testing results; available report-level sequencing metrics; and retrievable clinical information at the time of initial sampling, sequencing-result return, and management review.

After sequencing results were reviewed, the cases were analyzed descriptively because they showed clinically challenging interpretive relationships between routine testing and sequencing findings. These relationships included routine detection of one presumed pathogen with only low-support sequencing evidence for another organism, or routine-negative testing with a low-support sequencing signal of uncertain significance. Thus, the clinical indication for sequencing was suboptimal response after initial management, whereas the low-support or discordant nature of the sequencing findings was part of the retrospective interpretive analysis and was not the prospective indication for obtaining sequencing.

### 2.2 Data abstraction

For each case, we abstracted age, sex, specimen type, day of illness at sampling, major symptoms, routine laboratory report wording, routine test interpretation, sequencing summary metrics, top reported taxonomic hit, clinical status when sequencing results became available, whether management changed after result review, and short-term outcome. Management review or changes documented after sequencing-result return were abstracted descriptively only. The retrospective design did not allow independent adjudication of whether those changes were directly caused by sequencing, clinically appropriate or inappropriate, potentially unnecessary, or associated with improved outcomes. A structured case-level evidence summary integrating clinical context, routine-test classification, discordance scenario, sequencing metrics, quality-control (QC) and confirmation status, management impact, and interpretive category was prepared as Supplementary Table S1. Detailed report-level sequencing metrics for each case were summarized separately in Supplementary Table S2.

### 2.3 Specimen handling and processing

Upper respiratory swab specimens were initially collected by treating clinicians for routine respiratory testing during the first clinical and laboratory assessment. After routine testing, the corresponding swab specimens were frozen and archived. When clinical response after initial evaluation and treatment was considered suboptimal, the corresponding archived swab specimens were retrieved from storage for supplementary sequencing-based pathogen assessment. Therefore, sequencing was performed on archived corresponding swab material from the original clinical specimen rather than on newly collected supplementary swabs. Routine testing and sequencing were linked to the same original clinical specimen, although the retrospective records did not allow detailed reconstruction of the exact aliquot or residual material used for each routine assay.

For sequencing submission, the retrieved swab specimens were sealed, maintained in a frozen state on dry ice during transport, and delivered to Jilin Comatebio Biotechnology Co., Ltd. within 24 h. Detailed specimen-level information on the duration of frozen storage before retrieval for sequencing and the number of freeze-thaw events was not available in sufficient detail for quantitative analysis across cases. Therefore, specimen-handling information was described for methodological transparency, but storage duration and freeze-thaw variables were not used to infer quantitative differences in sequencing yield across cases.

### 2.4 Sequencing workflow and available report-level metrics

Sequencing-based respiratory pathogen analysis was performed using a provider-performed commercial ribonucleic acid (RNA) virome workflow at Jilin Comatebio Biotechnology Co., Ltd. The service was used as a supplementary pathogen-assessment workflow after suboptimal response to initial clinical evaluation and treatment. The authors did not receive documentation confirming whether this specific workflow had been validated under the Clinical Laboratory Improvement Amendments (CLIA), was Conformité Européenne-marked for *in vitro* diagnostic use (CE-IVD), was approved by the National Medical Products Administration (NMPA), or was provided for research use only; the workflow was not independently validated by the study team. No assay-specific published validation study for this exact provider-performed workflow was available to the authors. Therefore, the assay is described in this manuscript as a provider-performed commercial sequencing workflow rather than as an independently validated diagnostic test.

Across the full case series, report-level sequencing metrics were extracted from the commercial sequencing reports and summarized in Supplementary Table S2. These metrics included raw read pairs, clean read pairs, filtered read pairs, assembled contigs, retained pathogen-associated contigs, retained pathogen-associated contig length, pathogen-associated contig N50, maximum pathogen-associated contig length, read pairs mapped to pathogen-associated contigs, top-hit identity, aligned length, and top reported taxonomic hit. Integrated case-level interpretive summaries, including clinical context, routine-test classification, QC/confirmation status, specimen-matched negative-control status, provider-level QC context, and interpretive category, are provided in Supplementary Table S1. In this study, N50 denotes the contig length at which 50% of the total retained contig length is contained in contigs of that length or longer.

Provider workflow documentation was used to clarify method-level information. The documented RNA virome workflow described RNA extraction, reverse transcription, second-strand complementary DNA (cDNA) synthesis, cDNA fragmentation, library construction, and paired-end sequencing. Depending on specimen type and analytical purpose, the provider documentation described virus-like-particle enrichment and non-enrichment processing strategies. Sequencing was documented as being performed on an Illumina or BGISeq platform using paired-end 150-base-pair (PE150) reads; however, the exact sequencing instrument used for each case was not independently verifiable from the available records.

The provider documentation described quality filtering using fastp or Trimmomatic; subtraction of host and ribosomal RNA (rRNA) sequences using the Short Oligonucleotide Analysis Package aligner (SOAPaligner) or the Burrows-Wheeler Aligner (BWA) against host genomic sequences and the SILVA 132 rRNA database; and *de novo* assembly using metagenomic assemblers such as MEGAHIT, SPAdes, IDBA, metaSPAdes, or Trinity (6–8). The detailed workflow output available to the authors specified MEGAHIT version 1.1.2 for assembly, whereas complete per-case software implementation, full software versions, command-line parameters, and run-specific settings were not available for independent verification. Viral sequence identification and classification were described as CheckV-supported and based on average amino acid identity (AAI) and hidden Markov model (HMM) approaches (9), with DIAMOND-supported sequence comparison and annotation against a provider-built viral database (version 4). This database was documented as including checkv-db version 1.5, National Center for Biotechnology Information (NCBI) GenBank viral sequences, the Gut Virome Database (GVD), the Gut Phage Database (GPD), the Metagenomic Gut Virus catalogue (MGV), the Cenote Human Virome Database (CHVD), Riboviria, the International Committee on Taxonomy of Viruses (ICTV) Master Species List 38, and LucaProt-derived results. Read mapping to viral contigs was described as being performed using BWA, with alignments covering less than 80% of the read length removed.

Provider-reported quality-control procedures included batch-level extraction blanks, no-template controls, library blanks, negative-control threshold filtering, contamination-aware filtering, and manual review. However, some workflow components were proprietary or provider-controlled and could not be fully disclosed or reconstructed from the available records. Detailed raw batch-level QC reports, raw intermediate analytical outputs, complete per-case software versions and parameters, full taxonomic assignment thresholds, complete reference database release files, contig assembly parameters, provider-defined reporting cutoffs, and the provider's complete internal definition of retained pathogen-associated contigs were not available for independent reanalysis. Therefore, the present study distinguishes between provider-documented method-level workflow information, report-level sequencing metrics summarized in the Supplementary Tables, and unavailable provider-level workflow parameters. The study was designed to address an interpretive and reporting problem in routine clinical microbiology rather than to establish definitive etiologic adjudication, independent analytical validation, or comparative diagnostic performance.

### 2.5 Analytical validation, confirmation, and QC review

No independent analytical validation was performed as part of this retrospective descriptive case series. *Post hoc* orthogonal molecular confirmation could not be performed because no additional residual specimen material or extracted nucleic acid remained available after the archived swabs had been used for the sequencing workflow. Individual specimen-matched negative controls were not available for case-level interpretation. In addition, although the commercial provider supplied methodological clarification indicating the use of batch-level negative controls, negative-control threshold filtering, contamination-aware filtering, and manual review, detailed raw batch-level QC reports and raw intermediate analytical outputs were not available for independent reanalysis by the study team. Therefore, provider-level QC information was used only as contextual information and was not treated as a substitute for independent analytical validation, orthogonal confirmation, or specimen-matched negative-control review.

### 2.6 Definitions

Cases were considered clinically challenging when the relationship between routine testing and sequencing findings was discordant or otherwise difficult to interpret, including: (1) routine detection of one presumed pathogen with only low-support sequencing evidence for another pathogen; or (2) routine negative testing with low-support sequencing evidence for a respiratory pathogen.

In this manuscript, “retained pathogen-associated contigs” refers to contigs reported by the commercial provider as retained after its filtering, assembly, and taxonomic annotation workflow and associated with a reported pathogen or pathogen-related taxonomic hit. This was a provider-reported category rather than an independently redefined analytical category created by the study team. The provider's complete internal criteria for retaining pathogen-associated contigs, including any minimum contig length, identity threshold, coverage threshold, abundance threshold, k-mer requirement, or manual-review rule, were not available for independent verification. Therefore, this term does not imply independent reannotation, independent confirmation of pathogen origin, or definitive etiologic attribution by the study team.

Low-yield findings were operationally defined descriptively by sparse retained pathogen-associated contigs, low mapped read support to pathogen-associated contigs, limited aligned regions, or a combination thereof, rather than by a prespecified validated threshold (3, 5). We did not treat the commercial provider's reported taxonomic calls as definitive etiologic diagnoses, because clinical metagenomic findings require interpretation in relation to specimen type, sequencing support, contamination-control context, and confirmatory testing availability (1, 3–5). Instead, the study team retrospectively classified the reported findings as low-support signals based on available report-level evidence, including sparse retained pathogen-associated contigs, low mapped read support, limited aligned regions, discordance with routine testing, absence of documented orthogonal confirmation, absence of specimen-matched negative controls, and lack of independently reviewable raw batch-level QC outputs, as summarized in Supplementary Tables S1 and S2.

The term “low-support signal” therefore refers to an operational interpretive category used in this study, not to a validated diagnostic threshold. These findings were considered hypothesis-generating observations rather than disease-defining results. Selective confirmatory testing was considered appropriate when a residual specimen or nucleic acid material would have been available (3, 5).

### 2.7 Statistical analysis

Analysis was descriptive. Continuous variables are summarized as medians and ranges; categorical variables are presented as counts.

## 3 Results

### 3.1 Case inclusion and clinical characteristics

During the study period, 10 archived pediatric upper respiratory swab specimens were retrieved for supplementary sequencing and assessed for eligibility. All 10 met the inclusion criteria and were included in the final descriptive case series; no retrieved specimens were excluded (Supplementary Figure S1). The children were aged 6–10 years (median, 8 years), with equal numbers of boys and girls. Sampling occurred on illness days 3–6 (median, 5 days). All patients had fever; 5 had cough, 7 had sore throat, and 6 had nasal congestion or rhinorrhea. Nine specimens were nasopharyngeal swabs and one was a throat swab (Table 1).

**Table 1:** Clinical and routine-testing characteristics of 10 pediatric cases with low-yield or clinically challenging respiratory sequencing findings.

Case	Age, y	Sex	Specimen	Illness day	Key symptoms	Routine test classification	Top sequencing hit	Status when result returned	Documented management change	Outcome
1	7	M	Throat swab	4	Fever, cough, sore throat, nasal congestion/rhinorrhea	Influenza-positive	Rhinovirus B	Near recovery	No	Recovered
2	9	F	Nasopharyngeal swab	4	Fever, sore throat, nasal congestion/rhinorrhea	Routine test negative	Rhinovirus B	Near recovery	No	Recovered
3	8	M	Nasopharyngeal swab	6	Fever, cough	Influenza-positive	Mycoplasma-related hit	Severe/ongoing symptoms	Yes	Recovered
4	10	M	Nasopharyngeal swab	5	Fever, sore throat	Routine test negative	Rhinovirus	Near recovery	No	Recovered
5	7	M	Nasopharyngeal swab	4	Fever, cough, sore throat	Influenza-positive	Respiratory syncytial virus	Unchanged	Yes	Recovered
6	6	F	Nasopharyngeal swab	5	Fever, cough, nasal congestion/rhinorrhea	Influenza-positive	Rhinovirus	Improving	No	Recovered
7	7	M	Nasopharyngeal swab	3	Fever, sore throat, nasal congestion/rhinorrhea	Influenza-positive	Rhinovirus	Improving	No	Recovered
8	9	F	Nasopharyngeal swab	5	Fever, cough	Influenza-positive	Mycoplasma-related hit	Severe/ongoing symptoms	Yes	Recovered
9	9	F	Nasopharyngeal swab	6	Fever, sore throat, nasal congestion/rhinorrhea	Influenza-positive	Rhinovirus	Near recovery	No	Recovered
10	8	F	Nasopharyngeal swab	5	Fever, sore throat, nasal congestion/rhinorrhea	Influenza-positive	Respiratory syncytial virus	Unchanged	Yes	Recovered

All 10 cases involved archived upper respiratory swab specimens that had originally been collected for routine respiratory testing and were later retrieved for supplementary sequencing because of suboptimal clinical response after initial routine evaluation and treatment. After sequencing results were reviewed, the cases were analyzed because the relationship between routine laboratory reports and sequencing-based findings was discordant or otherwise clinically challenging to interpret. All patients had fever, and all ultimately recovered. F, female; M, male; y, years.

### 3.2 Routine testing and clinically challenging concordance

Routine respiratory testing was classified as influenza-positive in 8 cases and negative in 2 cases. Eight cases fell into the scenario in which routine testing suggested one presumed pathogen whereas sequencing yielded only a low-support signal for another, and two cases fell into the scenario in which routine testing was negative but sequencing produced a low-support pathogen signal of uncertain significance. Case-level concordance scenarios, sequencing metrics, QC/confirmation status, management impact, and interpretive categories are summarized in Supplementary Table S1.

### 3.3 Structured case-level evidence and sequencing support

To reduce purely anecdotal interpretation, each case was summarized using a structured evidence framework integrating clinical context, routine-test classification, discordance scenario, sequencing metrics, QC/confirmation status, management review, and interpretive category. Integrated case-level evidence summaries are provided in Supplementary Table S1, and detailed sequencing metrics are provided in Supplementary Table S2.

### 3.4 Sequencing summary findings

**Figure 1:**
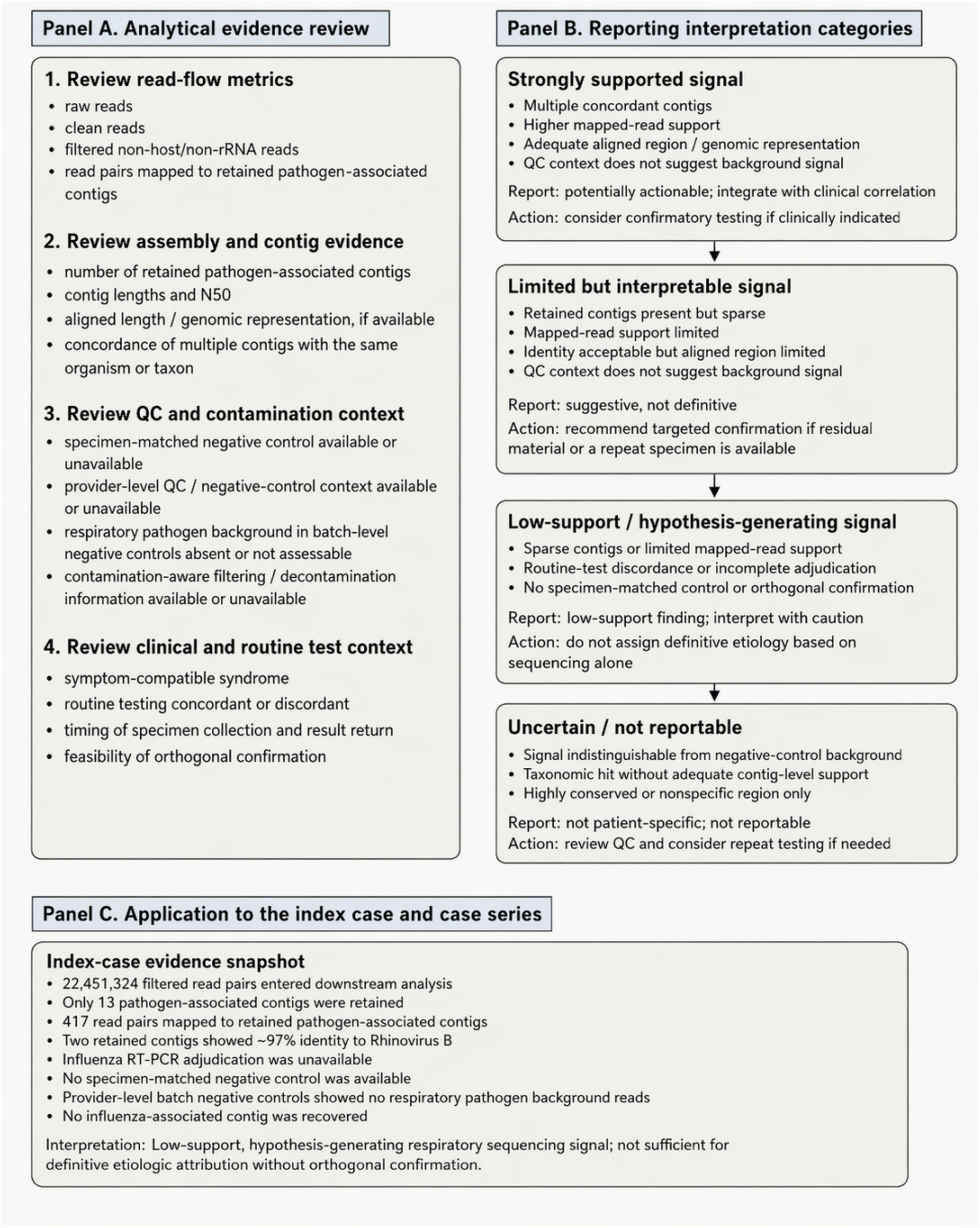
Preliminary pragmatic reporting framework for low-support respiratory sequencing findings in pediatric upper respiratory specimens. The figure summarizes a stepwise approach to interpreting and reporting low-yield respiratory sequencing results by integrating read-flow metrics, retained pathogen-associated contig characteristics, mapped read support, top-hit identity, routine-test concordance, availability of orthogonal confirmation, and provider-level QC/negative-control context. The present pediatric case series illustrates recurrent scenarios in which sequencing findings were analytically suggestive but not sufficiently supported for definitive etiologic attribution, thereby supporting cautious, structured laboratory reporting. This framework is intended as a cautious reporting aid and should not be interpreted as a validated diagnostic algorithm. Case-level evidence supporting this framework is summarized in Supplementary Table S1, and detailed report-level sequencing metrics are provided in Supplementary Table S2. N50, the contig length at which 50% of the total retained pathogen-associated contig length is contained in contigs of that length or longer; QC, quality control.

All 10 cases underwent sequencing-based analysis within the framework depicted in Figure 1. Across the series, raw sequencing output was substantial, with raw read pairs ranging from 49.6 to 70.0 million and filtered read pairs ranging from 20.1 to 30.3 million. However, pathogen-associated evidence was limited after filtering and assembly. Retained pathogen-associated contigs were sparse, with a median of 12.5 contigs (range: 5–17), median total retained pathogen-associated contig length of 10,056.5 base pairs (bp) (range: 9,856–13,121 bp), median contig N50 of 893.5 bp (range: 691–994 bp), and median maximum retained pathogen-associated contig length of 1,564 bp (range: 1,354–1,894 bp). Mapped read support to retained pathogen-associated contigs was low overall, with a median of 408.5 read pairs (range: 384–453). Median top-hit identity was 97.8% (range: 89.9%–99.3%), over aligned regions ranging from 691 to 994 bp. These report-level findings supported classification of the detections as low-support sequencing signals rather than definitive etiologic assignments.

Top low-support hits included rhinovirus/rhinovirus B in 6 cases, respiratory syncytial virus in 2 cases, and Mycoplasma-related hits in 2 cases. According to provider-level QC clarification, the corresponding sequencing batch(es) included negative controls, and no respiratory pathogen-related background reads were detected in those controls. These low-yield signals were therefore interpreted in the context of provider-reported negative-control filtering, statistical decontamination, and manual review, while recognizing that detailed raw QC reports were not available for independent reanalysis. These observations suggest that low-yield respiratory sequencing may generate analytically limited signals across more than one respiratory pathogen class, reinforcing the need to interpret such findings as reporting challenges rather than microbiologically definitive results. Detailed sequencing metrics for each case are provided in Supplementary Table S2, and integrated case-level evidence summaries, including interpretive categories, are provided in Supplementary Table S1.

The relatively narrow range of mapped read pairs across cases was interpreted cautiously. The provider's internal reporting thresholds, complete post-analytical filtering criteria, and raw intermediate analytical outputs were not available for independent verification; therefore, we could not determine whether the observed similarity reflected provider reporting thresholds, post-analytical filtering, rounding or reporting conventions, or other features of the commercial reporting workflow. Importantly, cases were not selected on the basis of mapped read counts, retained contig numbers, top-hit identity, aligned length, or a predefined sequencing-metric pattern, and no additional archived upper respiratory swab specimens retrieved for this supplementary sequencing series were excluded after application of the inclusion criteria. Accordingly, the relatively homogeneous mapped-read support should not be interpreted as evidence of a reproducible biological pattern or as evidence that the cases were selected to fit a predefined sequencing profile.

### 3.5 Index case

The index case remained the most analytically detailed specimen in the series. A 7-year-old boy with sore throat, cough, nasal congestion, and fever underwent throat swab sampling on illness day 4. Routine antigen testing suggested influenza infection, but influenza RT-PCR was unavailable. Sequencing generated 50,445,331 raw read pairs, 25,955,291 clean read pairs, and 22,451,324 post-filtered read pairs; *de novo* assembly yielded 4,911 contigs. Only 13 pathogen-associated contigs were retained, totaling 10,027 bp, with 417 read pairs mapped to retained pathogen-associated contigs overall. Two retained contigs showed high nucleotide identity (∼97%) to Rhinovirus B. No specimen-matched negative control was available for this case; however, provider-level clarification indicated that batch-level negative controls were included for the corresponding sequencing batch. No influenza-associated contig was recovered in the reported pathogen-associated contig set. This case therefore illustrated the central interpretive problem of the series: a routine laboratory result suggesting one presumed pathogen, alongside only low-support sequencing evidence for another respiratory pathogen.

### 3.6 Timing of result return and management impact

When sequencing results became available, 4 patients were already near recovery, 2 were improving, 2 were clinically unchanged, and 2 still had substantial symptoms, indicating that these low-yield findings were often more relevant to retrospective interpretation and reporting than to immediate acute management. A management review or change was documented after result review in 4 cases, whereas 6 had no documented change. However, because the records were retrospective and management decisions were made in real-world clinical contexts, the available data did not allow causal attribution of these changes to sequencing results alone. The appropriateness of the changes, including whether any treatment escalation, antimicrobial use, or other intervention was necessary or unnecessary, could not be independently adjudicated. Their relationship to clinical outcomes also could not be determined. All 10 patients ultimately recovered. Therefore, these observations should be interpreted as descriptive management context rather than evidence that low-yield sequencing results directly improved patient outcomes or provided measurable clinical benefit.

### 3.7 QC, confirmation status, and interpretive constraints

No case had documented orthogonal confirmation, and no independent analytical validation was performed on the reported sequencing hits. No specimen-matched negative control was available for individual case-level review. However, provider-level clarification indicated that the corresponding sequencing batch(es) included extraction blanks, no-template controls, and library blanks processed alongside clinical specimens, and that no respiratory pathogen-related background reads were detected in the negative controls. The provider also reported use of negative-control threshold filtering, statistical decontamination, and manual review for low-support hits. Nevertheless, detailed raw batch-level QC reports and raw intermediate analytical outputs were not retrievable for independent reanalysis, and the provider-level QC clarification could not be independently verified from raw QC files by the study team. Accordingly, the available provider-level QC information was considered supportive contextual information but did not substitute for orthogonal molecular confirmation, specimen-matched negative-control review, or independent analytical validation. These findings indicated that sequencing results required interpretation in the context of incomplete confirmation and limited independently reviewable QC documentation (3, 4).

## 4 Discussion

This study should be interpreted as a small, purposively selected, retrospective descriptive case series focused on the interpretation of low-yield and discordant respiratory sequencing reports in pediatric practice, not as a diagnostic validation study. Its purpose was not to prove etiologic causality, estimate diagnostic yield, or demonstrate that sequencing outperforms routine testing. Rather, the study illustrates how available report-level sequencing metrics, routine-test concordance, QC/negative-control context, and the availability of confirmatory testing can be integrated to support cautious laboratory reporting. Across the series, respiratory sequencing findings were analytically limited rather than microbiologically definitive, routine testing was often discordant with low-support sequencing signals, and results frequently became available when patients were already improving or near recovery. Provider-level QC clarification partly addressed contamination-related concerns by confirming the use of batch-level negative controls, absence of respiratory pathogen-related background reads in those controls, and contamination-aware filtering procedures. Nevertheless, the absence of *post hoc* orthogonal confirmation, the lack of specimen-matched negative controls, delayed result return, and the inability to independently review detailed raw QC outputs all support cautious interpretation and conservative reporting rather than definitive etiologic attribution (1–4).

The clinical utility of these low-yield sequencing results should therefore be interpreted cautiously. Although management review or change was documented in 4 of 10 cases after sequencing results became available, the retrospective records did not allow causal attribution of those changes to sequencing alone, nor could they establish that the changes were clinically appropriate, avoided unnecessary treatment, prevented antimicrobial overuse, or improved outcomes. Conversely, the available records also could not determine whether any management change represented potentially unnecessary treatment triggered by an uncertain low-support result. Some patients were already improving or near recovery when results returned, and all patients ultimately recovered. Accordingly, this study should not be interpreted as evidence that low-yield respiratory sequencing directly improved patient outcomes or meaningfully changed acute management. Rather, its primary contribution is diagnostic stewardship: it illustrates how laboratories and clinicians can recognize low-support sequencing signals, avoid treating uncertain taxonomic calls as disease-defining results, identify when confirmatory testing would be appropriate, and communicate uncertainty in a structured report.

These findings favor a conservative reporting approach in clinical microbiology. Provider-reported QC procedures help contextualize possible contamination, but they do not remove the need for caution when contig recovery and mapped-read support are limited. Reports should therefore summarize read-flow, contig burden, mapped read support, top-hit identity, routine-test concordance, QC/negative-control context, and availability of confirmatory testing. Accordingly, we treat these low-support detections as hypothesis-generating signals rather than disease-defining findings (3–5). This interpretation is consistent with a broader literature emphasizing that clinical mNGS results require cautious adjudication rather than automatic etiologic attribution. Reviews of clinical metagenomics have highlighted both the promise of broad pathogen detection and the hurdles created by analytical sensitivity, contamination, incomplete reference databases, reporting thresholds, and uncertain clinical relevance of low-abundance detections (15). Low-biomass sequencing studies further show that contaminant signals can disproportionately affect samples with limited microbial input, making negative controls, contamination-aware analysis, and transparent reporting essential for interpretation (16, 17). From a diagnostic stewardship perspective, sequencing results should be reported and acted on only in relation to the clinical syndrome, specimen type, pretest probability, routine-test concordance, and whether the result is likely to change management (18, 19).

Respiratory virus detection in children also requires caution because molecular positivity may occur in asymptomatic children or persist after recent infection, particularly for rhinovirus/enterovirus and some other respiratory viruses (20, 21). Similarly, Mycoplasma-related detections in pediatric upper respiratory specimens are difficult to interpret because current molecular or serologic tests may not reliably distinguish active infection from asymptomatic carriage or transient detection (11, 12). These considerations support our conservative reporting approach: low-support sequencing signals in upper respiratory specimens should be framed as hypothesis-generating observations, with targeted confirmatory testing recommended when residual material is available.

The Mycoplasma-related signals require especially conservative interpretation. In upper respiratory specimens from children, detection of Mycoplasma nucleic acid may reflect active infection, asymptomatic colonization or carriage, transient carriage after recent exposure, or contamination from sampling, environmental, reagent, or laboratory sources (11, 12). In the present series, the two Mycoplasma-related cases had limited genomic support and lacked orthogonal molecular confirmation, paired serology, specimen-matched negative controls, and independently reviewable raw QC outputs. Because current case-level evidence could not distinguish infection from carriage, transient detection, or contamination, these findings should not be used alone to establish etiologic causality or justify disease-defining interpretation. Instead, Mycoplasma-related low-support signals should prompt cautious reporting and, when residual material is available, targeted confirmation by polymerase chain reaction (PCR), quantitative PCR (qPCR), or paired serologic review before being incorporated into clinical decision-making.

The present study has several methodological and interpretive limitations. First, the sample size was very small, and the cases were clinically triggered rather than population-based or consecutively enrolled pediatric respiratory infection cases. All 10 archived pediatric upper respiratory swab specimens retrieved for supplementary sequencing between November 2025 and December 2025 were assessed for eligibility, met the inclusion criteria, and were included in the final descriptive case series. No additional archived upper respiratory swab specimens retrieved for this supplementary sequencing series were excluded after application of the inclusion criteria. However, sequencing was performed because treating clinicians were concerned about suboptimal response after initial evaluation and treatment. Therefore, the sampling strategy was shaped by clinical concern and is subject to substantial selection bias. The cases should not be interpreted as representing all pediatric respiratory infections, all children undergoing routine respiratory testing, or all respiratory sequencing tests during the same period. Consequently, the series cannot be used to estimate the prevalence, diagnostic yield, reproducibility, generalizability, or general distribution of low-yield sequencing findings in pediatric respiratory specimens.

Second, sequencing was performed through a provider-performed commercial RNA-virome workflow by Jilin Comatebio Biotechnology Co., Ltd. Although provider documentation supplied additional method-level information and report-level sequencing metrics were available for all cases in Supplementary Table S2, several technical, proprietary, regulatory, and validation-related details were not independently verifiable. These included the exact sequencing instrument used for each case, whether this specific workflow was CLIA-validated, CE-IVD-marked, NMPA-approved, or provided as a research-use-only service, the availability of any assay-specific published validation study for this exact workflow, the exact library-preparation kit, the complete extraction protocol for each clinical specimen, complete software and bioinformatics pipeline versions, command-line parameters, run-specific settings, complete reference database release files, full taxonomic assignment thresholds, contig assembly parameters, provider-defined reporting cutoffs, and the provider's complete internal definition of retained pathogen-associated contigs. As a result, the available sequencing information should be interpreted as provider-documented method-level information and report-level metrics, not as a fully reproducible or independently validated bioinformatics pipeline.

Third, no independent analytical validation was performed. No case had *post hoc* orthogonal molecular confirmation, and no specimen-matched negative control was available for individual case-level interpretation. Orthogonal PCR- or qPCR-based confirmation could not be performed because no additional residual specimen material or extracted nucleic acid remained available after the archived swabs had been used for the sequencing workflow. This limitation was particularly important for the Mycoplasma-related hits, because upper respiratory detection alone could not distinguish active infection from colonization, transient carriage, or contamination. Although the commercial provider reported batch-level negative controls, negative-control threshold filtering, contamination-aware filtering, and manual review, detailed raw batch-level QC reports and raw intermediate analytical outputs could not be retrieved for independent reanalysis. Provider-level QC information therefore helped contextualize contamination risk but did not substitute for independent analytical validation, orthogonal confirmation, or specimen-matched negative-control review.

Fourth, the relatively narrow range of mapped read support requires caution. Because provider-defined reporting thresholds, complete post-analytical filtering criteria, and raw intermediate analytical outputs were not available for independent verification, we could not determine whether the observed similarity reflected provider reporting thresholds, post-analytical filtering, rounding or reporting conventions, or other workflow-related factors. The cases were not selected according to mapped read counts, retained contig numbers, top-hit identity, aligned length, or any predefined sequencing-metric pattern, and no additional archived upper respiratory swab specimens retrieved for this supplementary sequencing series were excluded after application of the inclusion criteria. Therefore, the homogeneous mapped-read range should not be interpreted as a reproducible biological pattern.

Finally, the clinical utility of the low-yield sequencing results could not be established from the retrospective records. Although management review or change was documented in 4 cases, the study design did not allow causal attribution to sequencing alone, independent assessment of whether the changes were appropriate or potentially unnecessary, or determination of whether the changes improved outcomes. In particular, the available records could not determine whether any antimicrobial or other treatment decision represented an appropriate response to additional diagnostic information or potential overtreatment based on an uncertain low-support signal. Therefore, the present study should be interpreted as a diagnostic stewardship and reporting-focused case series rather than as evidence of direct clinical benefit. The proposed framework should be considered a preliminary pragmatic reporting aid rather than a validated diagnostic algorithm, and it requires further evaluation in larger, prospectively designed cohorts with standardized sequencing workflows, specimen-matched controls, orthogonal confirmation, and independently reviewable QC outputs.

The proposed framework is preliminary, but it is not based solely on narrative impression. It was constructed from five structured evidence domains available in this series: clinical context at the time of sampling and result return; concordance or discordance with routine respiratory testing; report-level sequencing support, including retained pathogen-associated contigs, mapped read support, top-hit identity, and aligned length; QC and negative-control context; and availability of orthogonal confirmation. These domains were used to classify reported detections as low-support signals, hypothesis-generating observations, or findings for which selective confirmatory testing would be appropriate if residual material were available.

Nonetheless, the study has practical value for pediatric respiratory diagnostic reporting and routine clinical microbiology practice. Even when low-yield sequencing findings do not immediately change management, case-level evidence summaries and provider-level QC context may help laboratories communicate uncertainty, identify when selective confirmatory testing would be appropriate, and avoid overinterpreting low-support signals. In this sense, Supplementary Table S1 and the proposed framework contribute to cautious retrospective interpretation and more disciplined reporting language (5, 10). Overall, low-yield respiratory sequencing findings in this small, purposively selected pediatric series are best understood as analytically limited, hypothesis-generating signals that require structured interpretation rather than definitive pathogen assignment. The proposed framework may help standardize cautious reporting language, but it requires further evaluation in larger, prospectively designed cohorts with standardized sequencing workflows, specimen-matched controls, orthogonal confirmation, and independently reviewable QC outputs.

## Data Availability

The original contributions presented in the study are included in the article and Supplementary Material. Further inquiries can be directed to the corresponding author. Raw sequence data are not readily available because of patient privacy considerations and institutional restrictions.
