# Supplementary material for: Low-yield respiratory sequencing in pediatric upper respiratory specimens: a case series and reporting framework
Source: Front Pediatr. 2026 Aug 5;14:1865124. doi: 10.3389/fped.2026.1865124 (PMC13486177; doi:10.3389/fped.2026.1865124)
Supplement: Supplementary file 1 [file Supplementaryfile1.docx]

***Supplementary Material***

**Supplementary Figure S1.** Case-identification flow diagram. The flow diagram summarizes archived pediatric upper respiratory swab specimens originally collected for routine respiratory testing and subsequently retrieved for supplementary sequencing after suboptimal clinical response. Ten archived specimens were retrieved between November 2025 and December 2025, 10 were assessed for eligibility, 0 were excluded after application of the inclusion criteria, and 10 were included in the final descriptive case series.
