# Supplementary material for: Low-yield respiratory sequencing in pediatric upper respiratory specimens: a case series and reporting framework
Source: Front Pediatr. 2026 Aug 5;14:1865124. doi: 10.3389/fped.2026.1865124 (PMC13486177; doi:10.3389/fped.2026.1865124)
Supplement: Supplementary file 2 [file Table1.docx]

***Supplementary Material***

**Supplementary Table S1. Structured case-level evidence summary for low-yield respiratory sequencing findings**

This supplementary table integrates routine-test context, sequencing metrics, and interpretive notes for all 10 pediatric cases described in the manuscript.

| **Case** | **Age, y** | **Sex** | **Specimen** | **Illness day** | **Key symptoms** | **Routine test** | **Top sequencing hit** | **Discordance scenario** | **Status at result return** | **Documented management change** | **Outcome** | **Raw reads** | **Clean reads** | **Filtered reads** | **Assembled contigs** | **Retained pathogen-associated contigs** | **Pathogen-associated contig length, bp** | **N50, bp** | **Max pathogen-associated contig, bp** | **Mapped read pairs** | **Identity, %** | **Aligned length, bp** | **Orthogonal confirmation** | **Specimen-matched negative control** | **Provider-level QC / negative-control context** | **Interpretive category** | **Evidence summary / comment** |
| --- | --- | --- | --- | --- | --- | --- | --- | --- | --- | --- | --- | --- | --- | --- | --- | --- | --- | --- | --- | --- | --- | --- | --- | --- | --- | --- | --- |
| 1 | 7 | M | Throat swab | 4 | Fever, cough, sore throat, nasal congestion/rhinorrhea | Influenza-positive | Rhinovirus B | Routine influenza-positive; sequencing yielded a low-support signal for another pathogen | Near recovery | No | Recovered | 50445331 | 25955291 | 22451324 | 4911 | 13 | 10027 | 911 | 1643 | 417 | 97 | 911 | No | No | Batch-level controls reported; no respiratory pathogen background reads detected; raw QC not independently reviewed | Low-support signal; interpret cautiously | Index case. Sparse retained pathogen-associated contigs and low mapped-read support. No influenza-associated contig was recovered in the reported pathogen-associated contig set; no orthogonal confirmation or specimen-matched negative control was available. |
| 2 | 9 | F | Nasopharyngeal swab | 4 | Fever, sore throat, nasal congestion/rhinorrhea | Routine test negative | Rhinovirus B | Routine negative; sequencing yielded a low-support pathogen signal | Near recovery | No | Recovered | 60504353 | 30488321 | 26584241 | 5125 | 15 | 10042 | 822 | 1562 | 395 | 98.2 | 822 | No | No | Batch-level controls reported; no respiratory pathogen background reads detected; raw QC not independently reviewed | Low-support signal; consider confirmatory testing if clinically indicated | Routine testing was negative, but sequencing showed a low-support Rhinovirus B signal with limited mapped reads and short aligned region. |
| 3 | 8 | M | Nasopharyngeal swab | 6 | Fever, cough | Influenza-positive | Mycoplasma-related hit | Routine influenza-positive; sequencing yielded a low-support signal for another pathogen | Severe/ongoing symptoms | Yes | Recovered | 52436251 | 26921480 | 23502668 | 18635 | 11 | 10784 | 935 | 1894 | 402 | 99.3 | 935 | No | No | Batch-level controls reported; no respiratory pathogen background reads detected; raw QC not independently reviewed | Low-support signal; selective confirmatory testing recommended | Clinically more symptomatic at result return. Sequencing suggested a Mycoplasma-related hit, but orthogonal confirmation was unavailable. Provider-level QC information was considered, although detailed raw QC outputs were not independently reviewable. |
| 4 | 10 | M | Nasopharyngeal swab | 5 | Fever, sore throat | Routine test negative | Rhinovirus | Routine negative; sequencing yielded a low-support pathogen signal | Near recovery | No | Recovered | 49625853 | 24673104 | 20149331 | 22186 | 10 | 9856 | 769 | 1421 | 388 | 96.5 | 769 | No | No | Batch-level controls reported; no respiratory pathogen background reads detected; raw QC not independently reviewed | Low-support signal; interpret cautiously | Routine testing was negative; sequencing yielded a limited Rhinovirus signal with low mapped-read support and no orthogonal confirmation or specimen-matched negative control. |
| 5 | 7 | M | Nasopharyngeal swab | 4 | Fever, cough, sore throat | Influenza-positive | Respiratory syncytial virus | Routine influenza-positive; sequencing yielded a low-support signal for another pathogen | Unchanged | Yes | Recovered | 53260141 | 27194225 | 22854941 | 21665 | 12 | 9998 | 896 | 1566 | 421 | 89.9 | 896 | No | No | Batch-level controls reported; no respiratory pathogen background reads detected; raw QC not independently reviewed | Low-support signal; selective confirmatory testing recommended | Low-support RSV signal with the lowest top-hit identity in the series. Result contributed to management review, but etiologic attribution remained uncertain. |
| 6 | 6 | F | Nasopharyngeal swab | 5 | Fever, cough, nasal congestion/rhinorrhea | Influenza-positive | Rhinovirus | Routine influenza-positive; sequencing yielded a low-support signal for another pathogen | Improving | No | Recovered | 54968336 | 28325412 | 23968393 | 23582 | 14 | 10071 | 954 | 1708 | 406 | 91.5 | 954 | No | No | Batch-level controls reported; no respiratory pathogen background reads detected; raw QC not independently reviewed | Low-support signal; interpret cautiously | Improving clinically when result returned. Sequencing provided only limited support for Rhinovirus in the setting of influenza-positive routine testing. |
| 7 | 7 | M | Nasopharyngeal swab | 3 | Fever, sore throat, nasal congestion/rhinorrhea | Influenza-positive | Rhinovirus | Routine influenza-positive; sequencing yielded a low-support signal for another pathogen | Improving | No | Recovered | 58772945 | 31498174 | 26479020 | 26540 | 17 | 10354 | 868 | 1487 | 453 | 98.6 | 868 | No | No | Batch-level controls reported; no respiratory pathogen background reads detected; raw QC not independently reviewed | Low-support signal; interpret cautiously | Highest mapped-read support in the series, but still low overall and without orthogonal confirmation or specimen-matched negative-control review. |
| 8 | 9 | F | Nasopharyngeal swab | 5 | Fever, cough | Influenza-positive | Mycoplasma-related hit | Routine influenza-positive; sequencing yielded a low-support signal for another pathogen | Severe/ongoing symptoms | Yes | Recovered | 67105284 | 35264851 | 30258482 | 60548 | 5 | 13121 | 994 | 1817 | 446 | 98 | 994 | No | No | Batch-level controls reported; no respiratory pathogen background reads detected; raw QC not independently reviewed | Low-support signal; selective confirmatory testing recommended | Very sparse retained pathogen-associated contigs despite reasonable top-hit identity. Mycoplasma-related signal should be considered hypothesis-generating rather than definitive. |
| 9 | 9 | F | Nasopharyngeal swab | 6 | Fever, sore throat, nasal congestion/rhinorrhea | Influenza-positive | Rhinovirus | Routine influenza-positive; sequencing yielded a low-support signal for another pathogen | Near recovery | No | Recovered | 52001227 | 26457903 | 22764617 | 24033 | 13 | 10013 | 891 | 1471 | 411 | 97.7 | 891 | No | No | Batch-level controls reported; no respiratory pathogen background reads detected; raw QC not independently reviewed | Low-support signal; interpret cautiously | Near recovery at result return. Sequencing showed a limited Rhinovirus signal in the context of influenza-positive routine testing. |
| 10 | 8 | F | Nasopharyngeal swab | 5 | Fever, sore throat, nasal congestion/rhinorrhea | Influenza-positive | Respiratory syncytial virus | Routine influenza-positive; sequencing yielded a low-support signal for another pathogen | Unchanged | Yes | Recovered | 70035484 | 33362751 | 29154006 | 38774 | 8 | 11960 | 691 | 1354 | 384 | 97.9 | 691 | No | No | Batch-level controls reported; no respiratory pathogen background reads detected; raw QC not independently reviewed | Low-support signal; selective confirmatory testing recommended | Low-support RSV signal with the shortest aligned region in the series. Result was clinically reviewed, but orthogonal confirmation and specimen-matched negative-control data were unavailable. |

**Notes.**

Low-support signal indicates sequencing evidence that was analytically limited and insufficient for definitive etiologic attribution.

Selective confirmatory testing recommended indicates cases in which targeted PCR/qPCR review would be appropriate if residual specimen or nucleic acid were available.

Orthogonal confirmation indicates whether post hoc PCR/qPCR or another independent confirmatory assay was documented.

Specimen-matched negative control indicates whether a control matched to the individual specimen was available for case-level review.

Provider-level QC / negative-control context summarizes information supplied by the commercial sequencing provider. Batch-level controls and contamination-aware filtering were reported, and no respiratory pathogen-related background reads were detected in negative controls; however, detailed raw batch-level QC outputs were not available for independent reanalysis.

N50 indicates contig N50.

This table integrates clinical context, routine-test classification, discordance scenario, sequencing interpretation, QC/confirmation status, provider-level QC context, and interpretive category for each case. “Low-support signal” indicates sequencing evidence that was analytically limited and insufficient for definitive etiologic attribution. “Selective confirmatory testing recommended” indicates cases in which targeted PCR/qPCR or another orthogonal assay would be appropriate if residual specimen or nucleic acid material were available.
