# Supplementary material for: Low-yield respiratory sequencing in pediatric upper respiratory specimens: a case series and reporting framework
Source: Front Pediatr. 2026 Aug 5;14:1865124. doi: 10.3389/fped.2026.1865124 (PMC13486177; doi:10.3389/fped.2026.1865124)
Supplement: Supplementary file 3 [file Table2.docx]

Supplementary Material

**Supplementary Table S2. Report-level sequencing metrics for 10 pediatric cases with low-yield respiratory sequencing findings**

| **Case** | **Raw read pairs** | **Clean read pairs** | **Filtered read pairs** | **Assembled contigs** | **Retained pathogen-associated contigs** | **Retained pathogen-associated contig length (bp)** | **Pathogen-associated contig N50 (bp)** | **Maximum pathogen-associated contig length (bp)** | **Read pairs mapped to pathogen-associated contigs** | **Top hit identity (%)** | **Aligned length (bp)** |
| --- | --- | --- | --- | --- | --- | --- | --- | --- | --- | --- | --- |
| 1 | 50,445,331 | 25,955,291 | 22,451,324 | 4,911 | 13 | 10,027 | 911 | 1,643 | 417 | 97.0 | 911 |
| 2 | 60,504,353 | 30,488,321 | 26,584,241 | 5,125 | 15 | 10,042 | 822 | 1,562 | 395 | 98.2 | 822 |
| 3 | 52,436,251 | 26,921,480 | 23,502,668 | 18,635 | 11 | 10,784 | 935 | 1,894 | 402 | 99.3 | 935 |
| 4 | 49,625,853 | 24,673,104 | 20,149,331 | 22,186 | 10 | 9,856 | 769 | 1,421 | 388 | 96.5 | 769 |
| 5 | 53,260,141 | 27,194,225 | 22,854,941 | 21,665 | 12 | 9,998 | 896 | 1,566 | 421 | 89.9 | 896 |
| 6 | 54,968,336 | 28,325,412 | 23,968,393 | 23,582 | 14 | 10,071 | 954 | 1,708 | 406 | 91.5 | 954 |
| 7 | 58,772,945 | 31,498,174 | 26,479,020 | 26,540 | 17 | 10,354 | 868 | 1,487 | 453 | 98.6 | 868 |
| 8 | 67,105,284 | 35,264,851 | 30,258,482 | 60,548 | 5 | 13,121 | 994 | 1,817 | 446 | 98.0 | 994 |
| 9 | 52,001,227 | 26,457,903 | 22,764,617 | 24,033 | 13 | 10,013 | 891 | 1,471 | 411 | 97.7 | 891 |
| 10 | 70,035,484 | 33,362,751 | 29,154,006 | 38,774 | 8 | 11,960 | 691 | 1,354 | 384 | 97.9 | 691 |

**Supplementary Table S2 legend.** This table summarizes report-level sequencing metrics available from the commercial sequencing reports, including raw read pairs, clean read pairs, filtered read pairs, assembled contigs, retained pathogen-associated contigs, retained pathogen-associated contig length, contig N50, maximum pathogen-associated contig length, mapped read pairs, top-hit identity, and aligned length. These metrics were used for descriptive interpretation only and do not represent independently validated diagnostic thresholds.
